# Calcaneal distraction vs. cast immobilization for the preoperative treatment of patients with Danis–Weber type C ankle fractures: a case–control study

**DOI:** 10.3389/fsurg.2024.1404746

**Published:** 2024-07-17

**Authors:** Bing Song, Jisheng Shi, Xiaohui Xu, Xiangfeng Hou, Jingkun Jia, Tongtao Pang

**Affiliations:** ^1^ Department of Orthopedic, Qilu Hospital of Shandong University Dezhou Hospital, Dezhou, Shandong, China; ^2^ Department of Emergency, Qilu Hospital of Shandong University Dezhou Hospital, Dezhou, Shandong, China

**Keywords:** ankle fracture, calcaneal distraction, cast immobilization, Danis–Weber type C ankle fractures, temporizing fixation

## Abstract

**Introduction:**

Ankle fractures require temporary fixation to allow swelling to subside prior to surgery; this is typically achieved using calcaneal distraction or cast immobilization. We compared the results of these methods in the treatment of Danis–Weber type C ankle fractures.

**Methods:**

This retrospective study analyzed the data of 86 patients with Danis–Weber type C ankle fractures, of whom 40 underwent calcaneal distraction and 46 underwent cast immobilization. Clinical measures including preoperative detumescence time, daily swelling value, skin condition, and pain, SF-36 Health Survey (SF-36) score and ankle scores were compared between the two groups.

**Results:**

Baseline characteristics did not differ significantly between the groups. Calcaneal distraction resulted in a lower preoperative detumescence time (6.22 ± 0.64 vs. 8.94 ± 0.82 days) and lower daily swelling values compared with cast immobilization, leading to a lower skin necrosis rate. Resting pain scores were significantly lower in the calcaneal distraction group than in the cast immobilization group at various postoperative time points (*P* < 0.05). Ankle function scores were higher in the calcaneal distraction group than in the cast immobilization group at 12 months postoperatively (*P* < 0.05), indicating improved outcomes. Additionally, the SF-36 quality of life scores of patients undergoing calcaneal distraction were notably superior to those in the cast immobilization group.

**Discussion:**

Calcaneal distraction is superior to cast immobilization in reducing soft tissue swelling, alleviating pain, and enhancing ankle function recovery in patients with Danis–Weber type C ankle fractures. Early calcaneal distraction upon hospital admission is recommended to optimize surgical outcomes.

## 1 Introduction

Ankle fractures are a common type of fracture, accounting for approximately 10% of all fractures (1–3). The soft tissues surrounding the ankle joint exhibit inherent fragility, rendering them susceptible to swelling, tension blisters, skin necrosis, and extrusion of fractured ends (4). Consequently, surgical intervention is often delayed to allow ankle swelling to subside, reducing the risk of surgical complications (5). Typically, a period of seven to ten days post injury is required to resolve soft tissue swelling prior to surgical intervention. During this period, effective temporary external fixation is necessary to stabilize the affected limb, alleviate patient discomfort, and promote the resolution of soft tissue swelling (6, 7).

Currently, the predominant methods for stabilizing the ankle joint involve immobilization in a cast, calcaneal distraction or external fixator. However, external fixators are less commonly utilized in clinical practice due to their high cost, which renders them unaffordable for many patients. Additionally, manual reduction of the ankle followed by cast immobilization can provide temporary relief from pain and fixation of the joint, ankle edema typically peaks three to five days post-fracture, at which point the tightness of the cast may compromise the distal blood circulation, precipitating the development of tension blisters. Furthermore, the ability to effectively monitor the condition of the skin at the fracture site is restricted (6, 8). Compared with cast immobilization, calcaneal distraction can better assist the reduction of swelling. However, the fixation device is unwieldy and can cause discomfort, neurovascular injury and psychological issues. Few studies have compared the two fixation methods for ankle fractures (6, 9–11). This study therefore retrospectively compared and analyzed the clinical effects of calcaneal distraction and cast immobilization in patients with Danis–Weber type C ankle fractures, with the aim of providing clinical data to aid in the selection of a perioperative temporary immobilization method in patients with ankle fractures.

## 2 Materials and methods

### 2.1 Patients

Patients who underwent open reduction and internal fixation of ankle fractures at Qilu Hospital of Shandong University Dezhou Hospital between January 2020 and December 2022 were considered for inclusion in this study. The inclusion criteria were as follows: (1) Danis–Weber type C fracture confirmed by imaging; (2) closed fracture; (3) absence of contraindications to surgery, such as skin and soft tissue injury at the fracture site upon admission; (4) patient election of open reduction and internal fixation surgery; and (5) complete clinical and follow-up data. The exclusion criteria were as follows: (1) open fracture, (2) pathological fracture, (3) severe physical or mental illness, and (4) incomplete clinical or follow-up data (12, 13).

This study was a single-center, retrospective cohort study. A total of 86 patients with ankle fractures met the eligibility criteria and were divided into two groups based on the type of temporary immobilization method used. The calcaneal distraction group consisted of 40 patients (23 with C1 fractures and 17 with C2 fractures), whereas the cast immobilization group comprised 46 patients (26 with C1 fractures and 20 with C2 fractures). Ethical approval for the study was obtained from the Ethics Committee of Qilu Hospital of Shandong University Dezhou Hospital, and the study adhered to the STROBE guidelines.

### 2.2 Preoperative preparation

All patients were treated with cold compression immediately after admission, and underwent preoperative radiography, computed tomography, and magnetic resonance imaging. Based on imaging data and various clinical indicators, a surgical plan was formulated following discussion among the medical team and with the patient.

Patients in the calcaneal distraction group underwent calcaneal distraction immediately after admission. With the patient in a recumbent position, the affected limb was lifted, keeping the foot perpendicular to the ground. The puncture point was the midpoint of the line between the tip of the medial malleolus and the posterior lower edge of the heel. Following the induction of local anesthesia, skeletal traction pins were inserted from the inside to the outside (to avoid damaging the posterior tibial nerve) and vertically into the calcaneus; the Kirschner wire was passed out from the lateral side of the calcaneus through the internal and external bone hammer. The traction force line should be consistent with the axis of the proximal end of the fracture, and the weight should be 1/10 to 1/12 body weight (14).

### 2.3 Surgical procedure

All patients received lumbar plexus block combined with infiltration anesthesia. Patients were placed in a supine or floating position based on their specific fracture morphology. Fractures of the lateral malleolus (fibula) were addressed through a standard lateral longitudinal incision followed by fixation using anatomical or 1/3 tubular steel plates. Fractures of the medial malleolus were managed with a curved anterior or posterior incision, with fixation achieved using tension screws. Posterior malleolar fractures were addressed through indirect restoration via a small anterior incision of the ankle joint, followed by fixation using tension screws placed anterior to posterior (15, 16).

### 2.4 Postoperative rehabilitation

The affected limbs were elevated after the operation and cold compression was applied for 24 h. Patient-controlled analgesia was provided initially, followed by routine postoperative analgesia; patients also received edema reduction, anti-inflammatory, and anticoagulant therapy. On the first postoperative day, ankle pump and isometric contraction of the quadriceps femoris exercises were initiated while the patient remained in bed. Machine-assisted joint activity training was initiated on postoperative day three (17, 18).

### 2.5 Observation indices

The main observation indices were skin condition at the surgical site, preoperative detumescence time, daily swelling value, resting pain score, perioperative complications,operation time and ankle functional outcome scores.

Swelling was evaluated by measuring the peripheral diameter at the swollen part of the affected limb upon admission and at one, three, and five days post-admission. The peripheral diameter of the ankle joint was measured at the tip of the medial malleolus. The swelling value was then calculated as the difference between the peripheral diameters of the affected and healthy limbs (19).

Ankle functional scores were based on subjective symptoms and function and were measured using the Foot and Ankle Outcome Score (FAOS) and Short Form 36 (SF-36) score. FAOS score has a maximum score of 100 points, and is divided into three components: mobility (20 points), function (30 points), and pain (50 points). Scores below 50 points indicate poor outcomes, scores between 50 and 74 points are considered qualified, scores between 75 and 89 points denote good outcomes, and scores ranging from 90 to 100 points signify excellent outcomes (20).

The SF-36 Health Survey (SF-36) measures the general quality of life. It is a multi-purpose, short-form health survey which contains 36 questions. It yields an eight-scale profile of scores as well as summary physical and mental measures (21).

### 2.6 Statistical analysis

All data were analyzed using SPSS software version 25.0 (IBM Corporation, Armonk, NY, USA). Measurement data were expressed as mean ± standard deviation. Normally distributed data were compared between the two groups using independent sample *t*-tests, and between multiple time points within groups using one-way analysis of variance. Count data were analyzed using the *χ*^2^ test or Fisher's exact test. *P* < 0.05 was considered statistically significant.

## 3 Results

### 3.1 Baseline data

Of the 86 patients with ankle fractures included in this study, 39 were male and 47 were female. There were no significant differences between the calcaneal distraction (*n* = 40) and cast immobilization (*n* = 46) groups in terms of age, sex, Danis–Weber classification, or cause of injury (*P* > 0.05), as shown in Table 1.

**Table 1:** Comparison of baseline data in the two groups.

Variable	Calcaneal distraction group (*n* = 40)	Cast immobilization group (*n* = 46)	*T* value/*χ*^2^ value*	*P* value
Age (years)	43.23 ± 13.24	44.73 ± 15.16	1.288	0.358
Sex			3.325*	0.529
Male	18	21		
Female	22	25		
Fracture type			10.388*	0.265
Type C1 (*n*)	23	26		
Type C2 (*n*)	17	20		
Cause of injury			8.796	0.328
Traffic accident	14	15		
Crush injury	4	6		
Ankle sprains	15	17		
Falling from height	6	8		

* means *χ*^2^ value.

### 3.2 Preoperative detumescence time and daily swelling value

The preoperative detumescence time of the calcaneal distraction group was significantly lower than that of the cast immobilization group (6.22 ± 0.64 vs. 8.94 ± 0.82 days; *P* = 0.031). There was no significant difference between the swelling value of the two groups upon admission, at 3.13 ± 0.91 cm in the calcaneal distraction group and 3.12 ± 0.84 cm in the cast immobilization group (*P* > 0.05). However, at one, three, and five days after admission, the swelling values of the calcaneal distraction group were 4.62 ± 0.48, 5.15 ± 0.37, and 2.69 ± 0.71 cm, respectively, significantly lower than those of the cast immobilization group, at 5.22 ± 0.74, 6.83 ± 0.77, and 5.23 ± 0.64 cm, respectively (*P* < 0.05), as shown in Table 2.

**Table 2:** Comparison of daily swelling value in the two groups (cm).

Groups	0d	1d	3d	5d
Calcaneal distraction group	3.13 ± 0.91	4.62 ± 0.48	5.15 ± 0.37	2.69 ± 0.71
Cast immobilization group	3.12 ± 0.84	5.22 ± 0.74	6.83 ± 0.77	5.23 ± 0.64
*T* value	1.452	4.721	5.105	7.849
*P* value	0.535	0.041	0.038	0.026

### 3.3 Resting pain score

Resting pain scores at 6, 12, 24, 48, and 72 h after calcaneal distraction or cast immobilization, as determined using a visual analog scale (VAS), are shown in Table 3. In the resting state, pain scores of both groups of patients gradually decreased over time. However, the resting pain scores of the cast immobilization group were significantly higher than those of the calcaneal distraction group at all time points (*P* < 0.05).

**Table 3:** Comparison of resting VAS score of patients in the two groups.

Groups	6 h	12 h	24 h	48 h	72 h
Calcaneal distraction group	6.72 ± 1.24	5.83 ± 1.56	5.51 ± 1.23	4.36 ± 1.35	3.02 ± 1.12
Cast immobilization group	6.66 ± 1.32	6.50 ± 1.21	6.31 ± 1.04	5.28 ± 1.16	4.53 ± 1.04
*T* value	2.125	2.271	2.317	2.013	3.193
*P* value	0.042	0.039	0.038	0.042	0.031

### 3.4 Perioperative complications

All patients exhibited satisfactory postoperative recovery. In the calcaneal distraction group, 32 patients maintained optimal skin integrity throughout the perioperative period, 5 developed tension blisters, and 3 and 1 patients experienced superficial and deep skin necrosis, respectively. In the cast immobilization group, 28 patients maintained optimal skin integrity, 11 developed tension blisters, and 4 and 3 patients experienced superficial and deep skin necrosis, respectively. The incidence of skin necrosis was therefore significantly lower in the calcaneal distraction group than in the cast immobilization group (*P* = 0.018). In the calcaneal traction group, 1 patient experienced a nail site infection, while in the cast immobilization group, 2 patients suffered from reduction loss. Additionally, 3 patients underwent reoperations in the cast immobilization group due to ankle stiffness resulting from malunion of the fracture.

### 3.5 Postoperative ankle score

The Foot and Ankle Outcome Score (FAOS) assessments conducted at the 12-month follow-up revealed significantly higher scores in the calcaneal distraction group compared to the cast immobilization group. Additionally, the SF-36 quality of life scores of patients undergoing calcaneal distraction were notably superior to those in the cast immobilization group, with statistically significant differences observed (*P* < 0.05). However, no significant difference in fracture healing time was observed between the groups (Table 4).

**Table 4:** Comparison of FAOS score, SF-36 score and fracture healing time in the two groups.

Type	Calcaneal distraction group	Cast immobilization group	*T* value	*P* value
FAOS score	85.62 ± 8.33	78.39 ± 7.27	8.23	0.036
SF-36 score	82.52 ± 10.23	73.75 ± 11.39	7.35	0.041
Fracture healing time (month)	3.74 ± 0.83	3.83 ± 1.27	2.231	0.339

## 4 Discussion

In 1972, Weber classified ankle fractures into three types based on Denis classification, delineating them by the location and shape of fibular fractures. Type A fracture involves a lateral malleolus fracture with the lower articular surface of the tibia or below it. Type B fracture is characterized by a lateral malleolus oblique fracture, where the fracture line starts from the anterior medial side and extends proximally to the posterior lateral side. Type C fractures encompass abduction type injuries, including lower tibiofibular ligament fracture and proximal fibular oblique fracture (C1 type), and abduction type injuries, involving fibular fracture closer to the side and wider interosseous membrane tear (C2 type). Type C fractures may also entail medial malleolus fractures or triangular ligament fractures, with all three types often accompanied by posterior malleolus fractures. Notably, type C fracture represents the most severe form of ankle joint fracture, often involving interosseous membrane tears and lower tibiofibular ligament injuries (22, 23). Owing to the thin soft tissue around the ankle joint and its poor blood flow, the capillaries of the surrounding soft tissue rupture after a fracture, the permeability of the vascular wall increases, and the intravascular fluid penetrates the tissue gap, resulting in soft tissue swelling. The swollen soft tissue further compresses blood vessels, causing reflux obstruction, tissue hypoxia, further swelling, blisters, and penetration of the skin by the broken ends of the fracture, leading to skin necrosis. Patients therefore require fixation to promote the resolution of ankle swelling (24–26).

In this study, the swelling values of the calcaneal distraction group were significantly lower compared to the cast immobilization group at one, three, and five days post-admission, with measurements of 4.62 ± 0.48, 5.15 ± 0.37, and 2.69 ± 0.71 cm respectively for the former, and 5.22 ± 0.74, 6.83 ± 0.77, and 5.23 ± 0.64 cm respectively for the latter. Additionally, the resting pain scores of the cast immobilization group were consistently higher than those of the calcaneal distraction group across all time points. These findings underscore the significance of calcaneal distraction in perioperative pain management and swelling reduction in ankle fractures. Cast immobilization stabilized the ankle joint, thereby preventing further displacement of the fractured ends and minimizing irritation to the surrounding soft tissues. This aids in the reduction in swelling. However, the traditional plaster casting process may exacerbate ankle joint swelling due to the heat released during plaster bandage hardening and shaping. Additionally, inadequate surgeon experience, as well as other factors, may result in uneven shaping of the plaster cast, leading to poor conformity with the ankle joint. In such cases, the cast may exert pressure on the ankle joint, exacerbating swelling (11, 27) and leading rapidly to tension blisters and superficial skin necrosis. In this study, the incidence of tension blisters and skin necrosis was significantly higher in the cast immobilization group than in the calcaneal distraction group. This was attributed to the continuous traction exerted by calcaneal distraction, which alleviates the compression of the surrounding soft tissues resulting from irregular fracture displacement and mitigates sustained stimulation of the adjacent blood vessels, nerves, and muscles. This traction facilitates the restoration of normal vascular function, enhances venous and lymphatic reflux, and effectively reduces soft tissue edema. Consequently, the preoperative detumescence time is shortened, thereby reducing the likelihood of soft tissue and skin necrosis.

Soft tissue swelling around ankle fractures often lasts for seven to ten days, delaying surgery; it is mainly resolved by absorption into the local capillary blood circulation. In this study, the detumescence time and the daily swelling values were lower in the calcaneal distraction group than in the cast immobilization group. This suggests that calcaneal distraction is more effective than cast immobilization at promoting detumescence of ankle joint fractures. Calf muscles can perform isometric contractions during traction. This prevents seepage from the peripheral capillaries while compressing the swelling, promoting its spread into the surrounding normal tissues, expanding the absorption area, and reducing the swelling of the soft tissue around the ankle joint and the adhesion and degeneration of the joint (7). Consistent with this, ankle scores 12 months postoperatively revealed superior outcomes in the calcaneal distraction group compared with the cast immobilization group. Moreover, calcaneal distraction demonstrated superior outcomes in terms of resting pain scores and postoperative ankle functional scores, as evidenced by higher FAOS and SF-36 scores at the 12-month follow-up. This highlights the potential of calcaneal distraction methods to enhance patient comfort and overall quality of life postoperatively.

For patients undergoing calcaneal distraction, early mobilization of the ankle joint is feasible because this technique effectively addresses tendon contractures, joint capsule limitations, and other soft tissue issues. Additionally, calcaneal distraction facilitates the initial reduction of fractures through ligament and joint capsule traction, thereby preventing postoperative ankle joint contracture and stiffness. Conversely, cast immobilization typically involves a seven to ten day fixation period, often resulting in postoperative ankle stiffness and ligament adhesions (28, 29). Notably, due to pronounced ankle joint swelling, the pain experienced by patients with fractures in this region tend to be higher than that experienced by patients with fractures elsewhere. Although using a cast effectively reduces patient discomfort and maintains mobility, it's challenging to monitor swelling, and regularly removing the cast becomes necessary. However, this process can accidentally shift the fracture ends. In our research, we observed that delaying the removal of bandages due to early-stage limb swelling led to significant blistering and discomfort in three patients. Furthermore, two patients in the cast immobilization group experienced a loss of reduction, and three patients required reoperations due to ankle stiffness resulting from improper healing of the fracture. These findings highlight the effectiveness of heel bone traction over external fixation with plaster casts in preventing ankle joint redislocation and stiffness.

During calcaneal distraction, the traction direction should be dynamically adjusted as needed, and the traction weight fine-tuned under fluoroscopic guidance. Bedside radiography enables precise preoperative anatomical reduction, ensuring optimal alignment. This not only facilitates edema reduction and prevents skin and soft tissue contractures, but also aids intraoperative maneuvers, thereby reducing the risk of forceful reductions during surgery. Proper nursing care of the skeletal traction pin insertion sites is essential for patients undergoing calcaneal distraction. The gauze should be replaced daily for the first two days to prevent blood clot formation. Subsequently, disinfection of the site every 3–4 h is necessary to keep the area clean and dry and minimize the risk of exudate accumulation. These sites increase the risk of infections, although no postoperative infections were observed in our study. Therefore, strict adherence to aseptic techniques during preoperative disinfection and preparation, and protection of the skeletal traction pin insertion sites, is imperative.

The study presents several limitations. Firstly, the absence of comparative analyses involving temporary fixation with external fixators arises from the limited clinical sample size of patients undergoing such procedures in our institution. This limitation curtails the comprehensive evaluation of various fixation modalities. Moreover, the retrospective, single-center design of this study introduces potential selection bias and constrains the generalizability of findings due to regional variations. Additionally, while the sample size meets statistical requirements, it may not sufficiently capture the diversity of patient demographics and treatment outcomes. The relatively brief 12-month follow-up duration further restricts the assessment of long-term treatment effects and complications, potentially overlooking crucial trends over extended periods. Furthermore, the study's failure to account for individual patient characteristics and external factors, such as trauma mechanism and surgical technique variations, could introduce confounding variables into the results. To address these limitations, we are currently planning a follow-up study explicitly designed to comprehensively compare the efficacy of the three fixation modalities under investigation. Moreover, necessitates future research adopting multicenter, large-sample randomized controlled trials with extended follow-up periods to enhance result generalizability and comprehensively evaluate treatment outcomes. Additionally, incorporating subgroup analyses based on individual patient characteristics and implementing measures to control external factors during study design and analysis could strengthen result validity. In summary, for patients with ankle fractures, early calcaneal distraction upon hospital admission is recommended to promote the resolution of soft tissue swelling, alleviate surgical challenges, and enhance postoperative ankle function recovery.

## Data Availability

The raw data supporting the conclusions of this article will be made available by the authors, without undue reservation.
